# Serology reveals heterogeneity of *Plasmodium falciparum* transmission in northeastern South Africa: implications for malaria elimination

**DOI:** 10.1186/s12936-017-1701-7

**Published:** 2017-01-26

**Authors:** Joseph Biggs, Jaishree Raman, Jackie Cook, Khumbulani Hlongwana, Chris Drakeley, Natashia Morris, Ishen Serocharan, Eunice Agubuzo, Philip Kruger, Aaron Mabuza, Alpheus Zitha, Elliot Machaba, Maureen Coetzee, Immo Kleinschmidt

**Affiliations:** 1 Faculty of Infectious and Tropical Diseases, London School of Hygiene and Tropical Medicine, London, UK; 2 Centre for Opportunistic Tropical and Hospital Infections, National Institute for Communicable Diseases, Johannesburg, South Africa; 3 Wits Research Institute for Malaria, School of Pathology, Faculty of Health Sciences, University of the Witwatersrand, Johannesburg, South Africa; 4 Institute for Sustainable Malaria Control, University of Pretoria, Pretoria, South Africa; 5 Tropical Epidemiology Group, Department of Infectious Diseases Epidemiology, London School of Hygiene and Tropical Medicine, London, UK; 6 School of Nursing and Public Health, University of KwaZulu-Natal, Durban, South Africa; 7 Health GIS Centre, South Africa Medical Research Council, Durban, South Africa; 8 Limpopo Provincial Malaria Control Programme, Polokwane, South Africa; 9 Mpumalanga Provincial Malaria Control Programme, Nelspruit, South Africa

**Keywords:** Malaria, Elimination, Serology, PfAMA-1, PfMSP-1_19_, Transmission, Heterogeneity, Hotspot, South Africa

## Abstract

**Background:**

It is widely acknowledged that modifications to existing control interventions are required if South Africa is to achieve malaria elimination. Targeting indoor residual spraying (IRS) to areas where cases have been detected is one strategy currently under investigation in northeastern South Africa. This seroprevalence baseline study, nested within a targeted IRS trial, was undertaken to provide insights into malaria transmission dynamics in South Africa and evaluate whether sero-epidemiological practices have the potential to be routinely incorporated into elimination programmes.

**Methods:**

Filter-paper blood spots, demographic and household survey data were collected from 2710 randomly selected households in 56 study wards located in the municipalities of Ba-Phalaborwa and Bushbuckridge. Blood spots were assayed for *Plasmodium falciparum* apical membrane antigen-1 and merozoite surface protein-1_19_ blood-stage antigens using an enzyme linked immunosorbent assay. Seroprevalence data were analysed using a reverse catalytic model to determine malaria seroconversion rates (SCR). Geospatial cluster analysis was used to investigate transmission heterogeneity while random effects logistic regression identified risk factors associated with malaria exposure.

**Results:**

The overall SCR across the entire study site was 0.012 (95% CI 0.008–0.017) per year. Contrasting SCRs, corresponding to distinct geographical regions across the study site, ranging from <0.001 (95% CI <0.001–0.005) to 0.022 (95% CI 0.008–0.062) per annum revealed prominent transmission heterogeneity. Geospatial cluster analysis of household seroprevalence and age-adjusted antibody responses detected statistically significant (p < 0.05) spatial clusters of *P. falciparum* exposure. Formal secondary education was associated with lower malaria exposure in the sampled population (AOR 0.72, 95% CI 0.56–0.95, p = 0.018).

**Conclusions:**

Although overall transmission intensity and exposure to malaria was low across both study sites, malaria transmission intensity was highly heterogeneous and associated with low socio-economic status in the region. Findings suggest focal targeting of interventions has the potential to be an appropriate strategy to deploy in South Africa. Furthermore, routinely incorporating sero-epidemiological practices into elimination programmes may prove useful in monitoring malaria transmission intensity in South Africa, and other countries striving for malaria elimination.

**Electronic supplementary material:**

The online version of this article (doi:10.1186/s12936-017-1701-7) contains supplementary material, which is available to authorized users.

## Background

Uninterrupted implementation of effective control measures since 2001 has substantially reduced malaria transmission intensity (MTI) in South Africa [1]. In 2012, with a national incidence below the World Health Organization (WHO) malaria elimination threshold of <one case per 1000 population at risk [2], the Malaria Directorate reoriented programme focus from malaria control to elimination. In support of this shift in focus, a strategic plan aiming to halt local malaria transmission within South African borders by 2018 is now in place [3]. To date, this elimination programme has made steady progress but still faces challenges; one acknowledged obstacle is the identification and targeting of malaria hotspots of transmission as a means of combating the disease [4].

The ability to stratify malaria risk and target areas with persistent, higher transmission intensities is integral to any effective elimination strategy [5, 6]. Several methods to determine MTI have been developed, although each is associated with its own set of shortcomings and challenges [7–9]. Health facility data, entomological inoculation rates (EIRs) and parasite prevalence are three of the most commonly used surrogate indicators of MTI. However, a reliance on the detection of parasitic material in either humans or mosquitoes reduces the sensitivity of these methods in unstable and low transmission areas, where parasite carriage is generally low [10–12].

Since malaria antibodies persist for extended periods [13], often irrespective of parasite presence, and can be detected using a high throughput quantitative enzyme-linked immunosorbent assay (ELISA), determining the prevalence of malaria antibodies is a cost-effective, sensitive option for assessing MTI in low-transmission settings [14–17]. Blood stage anti-malaria antibodies have been shown to be reliable markers of malaria exposure [18] and have been used in numerous sero-epidemiological studies investigating the level of, and changes in, MTI along with geographical patterns in malaria exposure [15, 19–23]. The main, serologically derived, measure of MTI in these studies is the seroconversion rate (SCR). The SCR is the average annual rate at which a population converts from being seronegative to seropositive to malaria antigen(s), and has been used in various geographical settings to accurately determine both current and historical changes in MTI [7, 10, 18, 19]. Across a range of geographical settings, the SCR has been shown to correlate well with both malaria incidence and EIRs [7, 24, 25], although this association has not been validated among very low transmission settings where few serological, entomological and health metric surveys have been conducted simultaneously.

The study reported here forms part of a larger cluster, randomized, clinical trial investigating the efficacy of a targeted method of vector control delivery in response to passively detected cases [26]. This baseline, cross-sectional, seroprevalence survey was undertaken to determine the level and spatial characteristics of malaria transmission, as well as provide insights into the risk factors associated with malaria exposure in northeastern South Africa.

## Methods

### Study site location

**Fig. 1:**
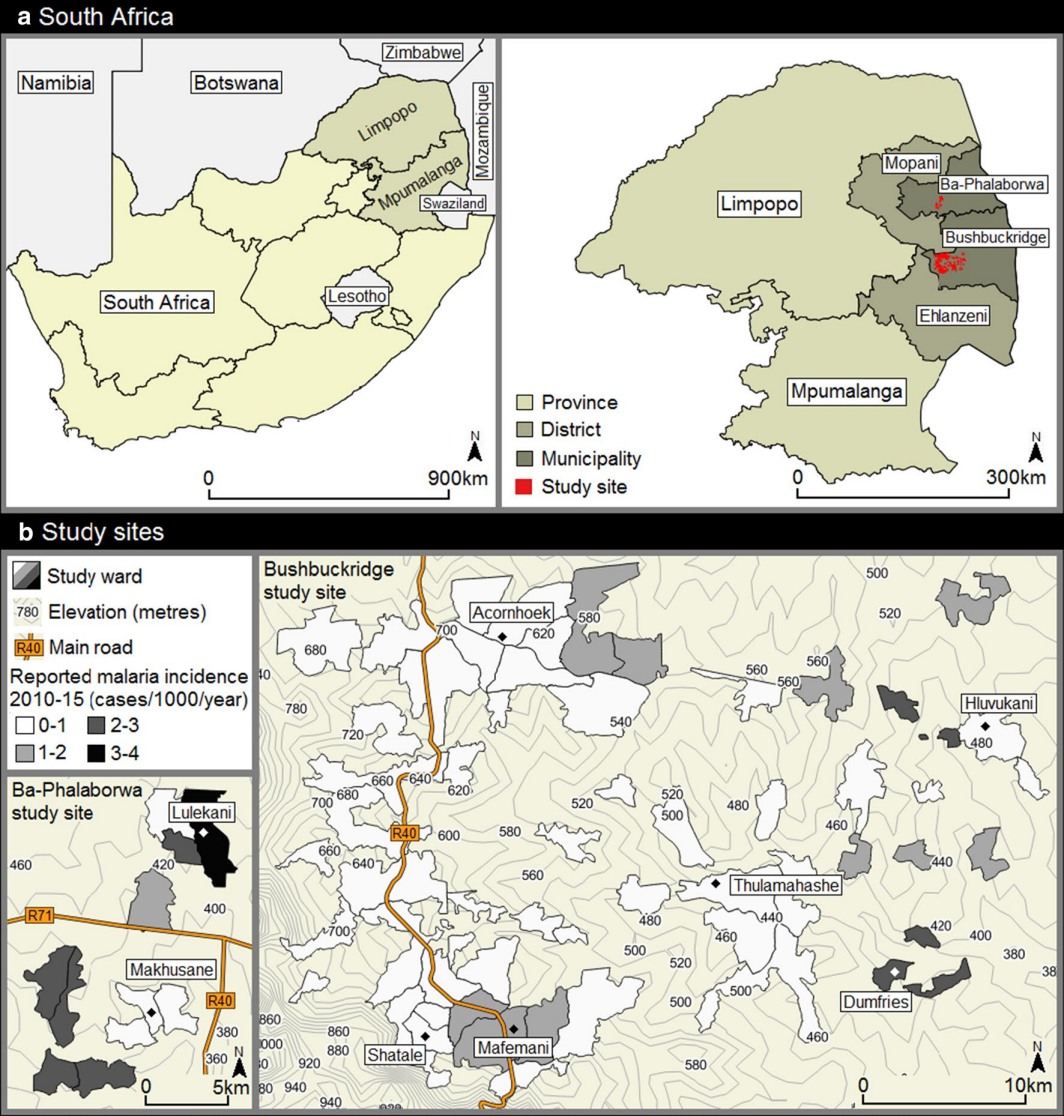
Location of the study sites situated within the municipalities of Ba-Phalaborwa and Bushbuckridge in northeastern South Africa. Passively reported malaria incidence data was obtained from health facilities between 2010 and 2015 then averaged among study site wards [43]

The study was conducted in the municipalities of Ba-Phalaborwa and Bushbuckridge, situated in the malaria-endemic provinces of Limpopo and Mpumalanga, South Africa, respectively (Fig. 1). Malaria transmission occurs predominately during the summer months from September to May [27], with the dominant parasite and vector being *Plasmodium falciparum* and *Anopheles arabiensis*, respectively [28]. The primary vector control intervention is annual generalized indoor residual spraying (IRS) of households in all areas in which malaria cases occur, using pyrethroids or dichlorodiphenyltrichloroethane (DDT) [1].

### Data collection

For the purposes of the targeted IRS, cluster randomized trial, the study area was divided into 56 study wards (13 in Ba-Phalaborwa, 43 in Bushbuckridge) (Fig. 1). Health facilities which operated as passive reporting systems in these wards, accumulated the number of reported malaria cases between 2010 and 2015, which enabled historical incidence to be calculated in the study area. Following collection over 5 years, historical malaria incidence was then averaged in each study ward. Between April and June 2015, a cross-sectional knowledge, attitudes and practices (KAP) and serological survey was undertaken among 60–80 randomly preselected households in each study site ward. Study personnel visited each of the selected households, obtained consent, then administered questionnaires concerning IRS practices, travel history and recent outdoor activity to the head of each household or their representative. Households with no adults present were excluded from the survey. Answers to survey questions were referred to the other consenting individuals residing in the household. Finger-prick dried blood spots (3MM Whatmann Paper) were collected from consenting adults (aged >18 years) and assenting children (aged >2–18 years) along with basic demographic information (age, gender education, employment status). Children under 2 years old were excluded from the survey. Parasite prevalence was not investigated in this study due to the very low reported mean incidence in the region (approximately one per 1000 per annum), which makes prevalence surveys impractical.

### Assay of *P. falciparum* antibodies

Bloodspots were stored at 4 °C as described previously [25]. Sera, eluted from the filter paper blood spots, were subjected to a previously described quantitative indirect ELISA to detect IgG antibodies to blood stage *P. falciparum* apical membrane antigen-1 (PfAMA-1) and merozoite surface protein-1_19_ (PfMSP-1_19_) antigens [25]. In short, antibodies in 3-mm circular cuttings from filter paper blood spots were eluted in a 1/200 dilution of reconstitution buffer (phosphate buffer saline + 0.5% Tween-20 + 0.05% sodium azide). Sera were then assayed in duplicate for antibodies against both PfAMA-1 and PfMSP-1_19_ in 1/2000 and 1/1000 dilutions, respectively, to obtain two optical density (OD) values per sample. A serial dilution of pooled sera from a malaria hyperendemic African region was used as a positive control to standardize OD values.

### Statistical analysis

Duplicate sample OD values were averaged and normalized against a positive control curve generated from hyperimmune sera. Sample OD values that differed more than 50% were dropped, and when possible repeated. Samples were then dichotomized as either seropositive or seronegative using a two component finite mixture model with five standard deviations as described in [7, 25]. Seropositive was defined as being positive to either PfAMA-1 and/or PfMSP-1_19_. SCRs were obtained from age-specific seroprevalence curves generated from reverse catalytic models, while age-adjusted antibody responses for PfAMA-1 and PfMSP-1_19_ were derived from log-transformed, normalized OD values as previously described in [15, 29]. Age-adjusted antibody responses were then averaged per household and categorized, based equally around the median, as ‘lower than average’, ‘average’, ‘slightly higher than average’, ‘higher than average’, and ‘much higher than average’ to generate an antibody response heat map. Study ward percentage seroprevalence corresponds to the percentage of sampled seropositive households (households that contained at least one member seropositive to PfAMA-1 and/or PfMSP-1_19_) in each ward.

Statistical analysis was performed using Stata 13.0 (Stata Corp, College Station, TX, USA) to identify potential risk factors associated with *P. falciparum* exposure among sampled participants. Odds ratios (ORs) associated with being seropositive to either antigen were derived from random effects logistic regression models which adjusted for correlation at the study ward level. Correlation was adjusted for at the study ward level, opposed to the household level, as any correlation at household level is reflected in the model. Correlation at ward level therefore provides a more conservative assessment of significance. Adjusted odds ratios (AORs) were derived using a multivariable model, including the following covariates: age, gender, education status, employment status, IRS practices, travel history, night-time outdoor activity, elevation, and study site (Ba-Phalaborwa and Bushbuckridge). Each statistically significant multivariate model was developed using the forward stepwise approach which compared multivariate models to the most significant univariate model using p-values derived from likelihood ratio tests.

### Geospatial analysis

Household elevation was determined using point sampling analyses in ArcGIS (v10.3.1). Sampled households with corresponding GPS coordinates were provided with elevation values derived from a 90-m resolution raster elevation data file (DIVA-GIS). Using the same raster elevation data file, average study ward elevation was calculated using zonal statistical methods in ArcGIS (v10.3.1). For geospatial analysis of seroprevalence, households containing only seronegative individuals, at least one seropositive individual as well as household averaged, age-adjusted antibody responses to *P. falciparum* PfAMA-1 and PfMSP-1_19_ were geographically plotted using ArcGIS software (v10.3.1). The spatial software SaTScan (v.9.4.2) was used to detect clusters of seropositive households and higher than average age-adjusted antibody responses to PfAMA-1 and PfMSP-1_19_. In order to detect clusters of seropositive households (households with at least one seropositive individual), seropositive households were used as cases, while seronegative households were used as controls using the Bernoulli model. Higher than average, age-adjusted PfAMA-1 antibody responses were detected using the Normal model. The scan statistic involves a scanning window, which enumerates both the observed and expected cases (seropositive households or higher than average individual age adjusted responses) across the study area to calculate non-overlapping, statistically significant (p < 0.05) clusters with a maximum set radius of 3 km.

## Results

### Study enrolment

Of the 3522 visited households, 76.9% (2710) agreed to participate in the survey. Within these households, 56.7% (4948/8728) of the eligible participants were present and consented to filter-paper blood spot and demographic data collection. Among the non-consenting participants, 42.5% (425/999) refused without providing a reason. Serological analysis was successfully conducted on 97% (4783/4948) of the blood samples that were correctly labelled. Once assayed, 94.1% (4499/4783) of the serological data correctly matched with demographic data to enable serological analysis. This merged data then matched with 99.5% (4477/4499) of the survey data and were subjected to further statistical and geospatial analysis.

### Population demographics

Adult females were over-represented in this study with 75% (2651/3521) of those aged over 18 years being female and only 20% (956/4477) of all participants aged between two and 17 years. Unemployment was high among the working-age population with 85% (2601/3057) of those aged between 18 and 65 years reporting as unemployed. Formal education was limited among the adult population with 40% (1401/3521) of the study participants over 18 years having received no formal secondary education. Night-time outdoor activity was uncommon with only 21% (945/4477) of the surveyed population reported undertaking night-time outdoor activities the evening before the survey. Only 1.5% (65/4477) of the surveyed individuals reported travelling outside South Africa in the past 6 months. Lastly IRS activity was not widespread among the randomly selected households, only 41% (1109/2698) of households reported receiving IRS during the previous malaria season (Table 1).

**Table 1:** Demographic characteristics and risk factors associated with *P. falciparum* seroprevalence

Risk factor	Total		*P. falciparum* seropositive			Unadjusted			Adjusted		
	%n	n	%n	n	%n 95% CI	OR^a^	95% CI	*p* value	OR^b^	95% CI	p-value
Age (years)											
2–5	8.8	394	4.6	18	2.9–7.1	0.21	0.12–0.35	<0.001	0.22	0.13–0.38	<0.001
6–15	10.7	481	10	48	7.6–13.0	0.48	0.34–0.69	<0.001	0.52	0.36–0.75	0.001
16–40	45.8	2050	12.8	263	11.5–14.4	0.71	0.58–0.88	0.002	0.71	0.58–0.89	0.002
≥ 41	34.7	1552	17.1	266	15.4–19.1	1			1		
Gender											
Male	30.3	1357	10.7	145	9.2–12.4	0.67	0.54–0.84	<0.001	0.77	0.62–0.97	0.057
Female	69.7	3120	14.4	450	13.2–15.7	1			1		
Education status											
Primary and below	49.5	2218	13.6	302	12.3–15.1	1			1		
Secondary and above	50.5	2259	13	293	11.7–14.4	0.89	0.73–1.08	0.239	0.72	0.56–0.95	0.018
Employment status											
Unemployment	86.7	3881	13.1	508	12.1–14.2	0.36	0.11–1.19	0.094			
Some employment	0.5	21	19.1	4	7.67–40.0	1					
Full employment	12.8	575	14.4	83	11.8–17.5	0.42	0.12–1.44	0.168			
Household IRS between Aug 2014 and Feb 2015											
Yes	45.4	2034	11.8	239	10.4–13.2	0.93	0.73–1.18	0.537			
No	53.1	2375	14.8	351	13.4–16.3	1					
Not sure	1.5	68	7.4	5	3.18–16.1	1.05	0.40–2.81	0.916			
Travel outside S. Africa in the past 6 months											
Yes	1.5	65	21.5	14	13.3–33.0	1					
No	98.5	4412	13.2	581	12.2–14.2	0.63	0.31–1.26	0.190			
Outdoor nightime activity last night											
Yes	21.1	945	12.4	117	10.4–14.6	1					
No	78.9	3532	13.5	478	12.4–14.7	0.90	0.68–1.19	0.464			
Household elevation (m)											
350–450	41.4	1855	9.1	168	7.8–10.5	0.57	0.32–0.99	0.057			
451–550	18.3	818	18.1	148	15.6–20.9	1					
551–650	29.0	1296	18.1	235	16.1–20.3	1.06	0.64–1.77	0.823			
651–750	11.4	508	8.7	44	6.5–11.4	1.01	0.49–2.08	0.969			
Study site											
Ba-Phalaborwa	35.3	1580	6.8	107	5.6–8.1	0.23	0.09–0.63	0.004	0.26	0.10–0.74	0.012
Bushbuckridge	64.7	2897	16.9	488	15.5–18.2	1			1		
Total	100	4477	13.3	595	12.3–14.3						

Seropositivity to either PfAMA-1 and/or PfMSP-1_19_ across the study sites of Ba-Phalaborwa and Bushbuckridge. Individual level data: age, gender, education status and employment status. Household level data: household IRS between August 2014 and February 2015, travel outside South Africa in the past 6 months, outdoor activity last night and household elevation

^a^Adjusted for correlation at the study ward level

^b^Adjusted for Age, gender, study site and correlation at the study ward level

### *Plasmodium falciparum* antibody responses and associated risk factors

Seropositive cut-offs corresponding to the OD values of 0.067 and 0.103 for PfAMA-1 and PfMSP-1_19_, respectively, dichotomized the sample population as seropositive or seronegative to *P. falciparum.* Overall antibody responses to both PfAMA-1 and PfMSP-1_19_ were low among the 4499 study participants as no OD value exceeded a value of 0.5 and many seropositive OD values, particularly among those under 10 years, were just above the seropositive thresholds (Additional file 1). Approximately 13% (587/4499) of all sampled individuals were seropositive to PfAMA-1 and/or PfMSP-1_19_, with 9% (403/4499) and 6% (279/4499) seropositive to just PfAMA-1 and PfMSP-1_19_ antigens, respectively, (Additional file 2).

Seroprevalence increased with age across both study sites. Statistically, individuals aged between 2 and 5 years were less likely to be *P. falciparum* seropositive compared to individuals aged ≥41 years (AOR 0.22, 95% CI 0.13–0.38, p < 0.001) (Table 1). *P. falciparum* seroprevalence was lower among individuals who attained formal secondary education or above than those who achieved up to formal primary education (AOR 0.72, 95% CI 0.56–0.95, p = 0.018) (Table 1). There was weak evidence for an association between *P. falciparum* seroprevalence and being female, but no evidence for an association with employment status, recent IRS, recent travel outside of South Africa, night-time outdoor activity or household elevation (Table 1). Furthermore, no statistical association was observed between these risk factors and seroprevalence to just AMA-1 or MSP-1_19_ (Additional file 3).

### *Plasmodium falciparum* transmission intensity and spatial distribution

**Fig. 2:**
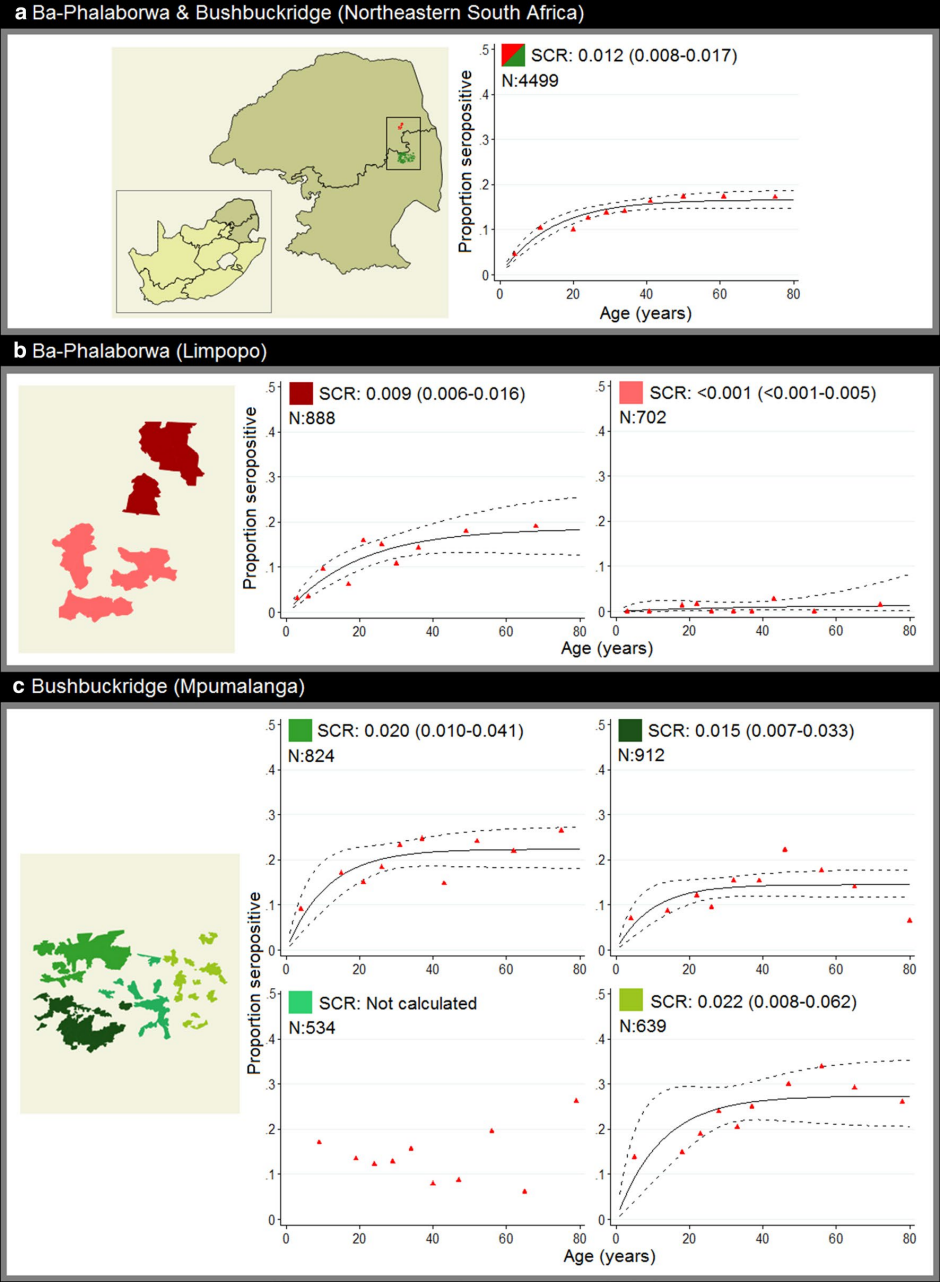
*Plasmodium falciparum* age-seroprevalence curves for the entire study site (**a**) and distinct geographical regions within each study site (**b**, **c**). Seroprevalence curves, fitted by maximum likelihood, represent the average annual rate at which this population become seropositive to either PfAMA-1 and/or PfMSP-1_19_ characterized by a seroconversion rate (SCR). *Red triangles*: observed age-seroprevalence, *solid lines*: predicted seroprevalence and *dotted lines*: predicted seroprevalence upper and lower 95% confidence intervals. N: sample size

MTI, as expressed by SCR, was estimated at 0.012 per annum across the entire study site (95% CI 0.008–0.017) (Fig. 2a). SCRs for separate antigens: PfAMA-1 and PfMSP-1_19_ equated to 0.011 (95% CI 0.007–0.017) and 0.003 (95% CI 0.002–0.005), respectively, per year (Additional file 2). MTI however, was not uniform across the study site, as SCRs corresponding to geographical distinct regions (grouped study site wards) varied considerably, ranging from <0.001 (95% CI <0.001–0.005) to 0.022 (95% CI 0.008–0.062) per annum (Fig. 2b, c). In the Ba-Phalaborwa study site alone, two regional SCR values were significantly different, equalling <0.001 (95% CI <0.001–0.005) and 0.009 (95% CI 0.006–0.016) per year (Fig. 2b).

**Fig. 3:**
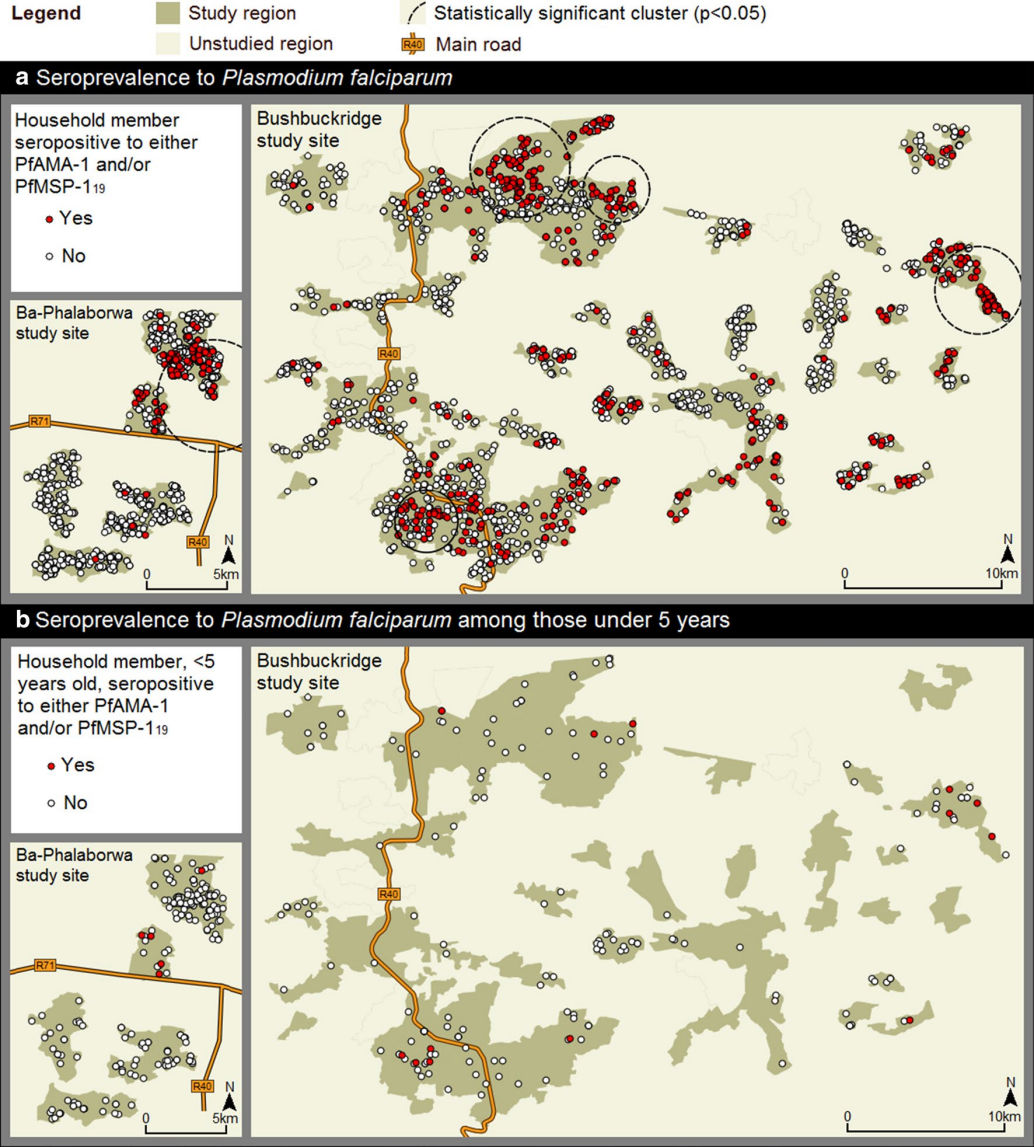
Spatial analyses of household *P. falciparum* seroprevalance across the Ba-Phalaborwa and Bushbuckridge study sites. **a** Spatial distribution of households containing ≥1 PfAMA-1 and/or PfMSP-1_19_ seropositive individual(s). SaTScan™ derived statistically significant (p-values <0.05) clusters of seropositive households reveal regions where there are a higher number of seropositive households than expected. **b** Spatial distribution of households containing ≥1 PfAMA-1 and/or PfMSP-1_19_ seropositive individual(s) aged 5 years and under

**Fig. 4:**
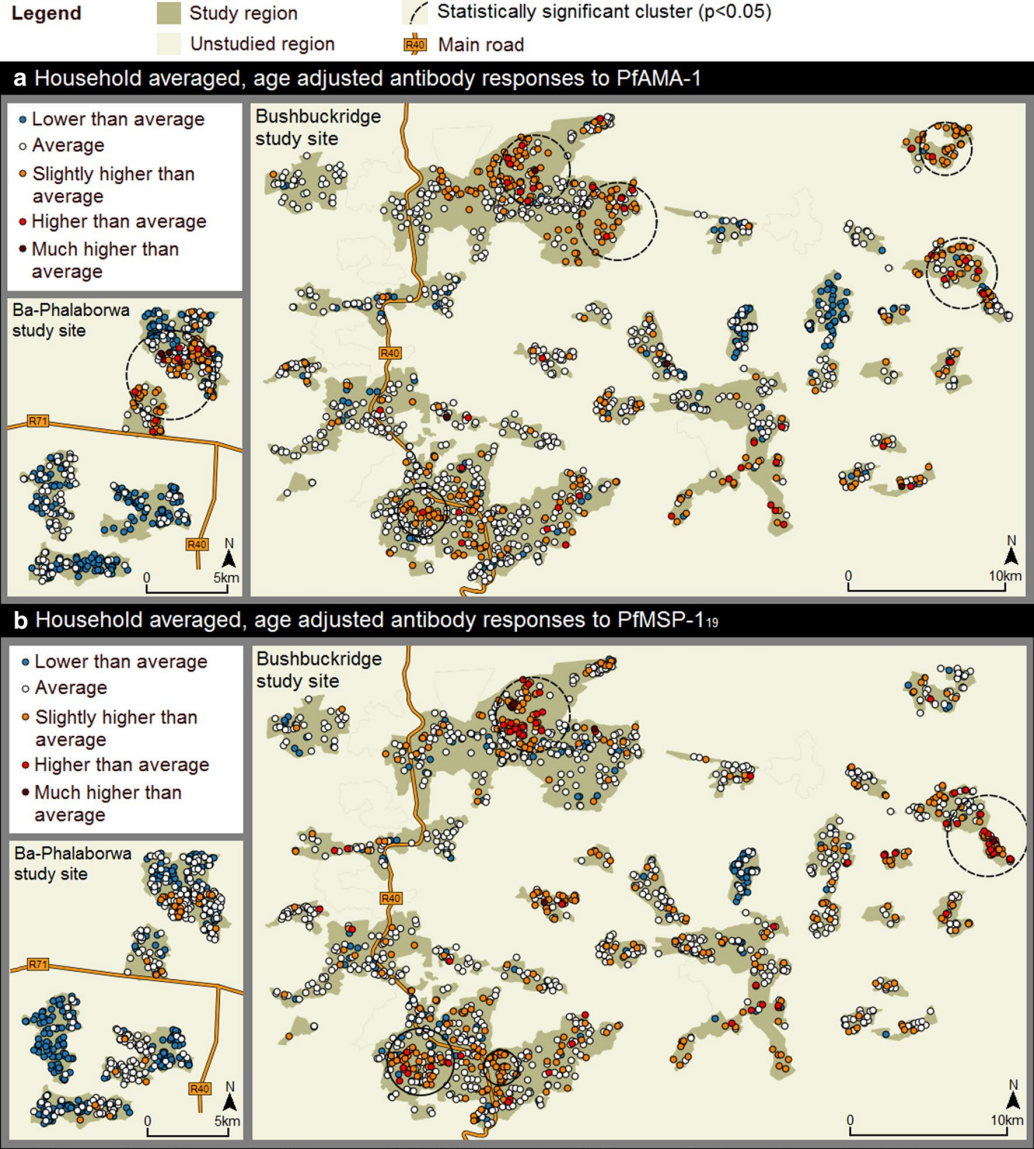
Spatial analyses of household-averaged, age-adjusted antibody responses to PfAMA-1 (**a**) and PfMSP-1_19_ (**b**) across the Ba-Phalaborwa and Bushbuckridge study sites. Age-adjusted antibody responses were derived from log-transformed PfAMA-1/PfMSP-1_19_ normalized OD values adjusted at 30 years. The resultant residual values were categorized as: as ‘lower than average’ (−2.370 to −0.499), ‘average’ (−0.500 to 0.500), ‘slightly higher than average’ (0.501–1.250), ‘higher than average’ (1.251–2.000) and ‘much higher than average’ (2.001–2.936). Statistically significant clusters (p-values < 0.05) of higher than average age-adjusted PfAMA-1/PfMSP-1_19_ antibody responses are also shown

Across both study sites, five statistically significant (p < 0.05) clusters of seropositive households (households containing at least one seropositive individual) were detected (Fig. 3). Clusters of higher than average, age-adjusted antibody responses to both PfAMA-1 and PfMSP-1_19_ were revealed in similar locations to clusters of seropositive households (Fig. 4). In total, 4/5 and 3/4 clusters of higher than average, age-adjusted antibody responses to PfAMA-1 and PfMSP-1_19_, respectively, spatially overlapped with clusters of seropositive households. This spatial overlap was less apparent between age-adjusted antibody responses to either PfAMA-1 or PfMSP-1_19_. In Ba-Phalaborwa, a cluster of higher than average, age-adjusted antibody responses to PfAMA-1 was identified but not to PfMSP-1_19_. Unlike seroprevalence among all ages, malaria seroprevalence in children under 5 years showed no statistical evidence (p > 0.05) of clustering. Nonetheless most children under 5 years were surveyed in houses situated in clusters of seropositive households containing all ages (Fig. 3a, b).

### Ward-level malaria incidence, elevation and *Plasmodium falciparum* seroprevalence

**Fig. 5:**
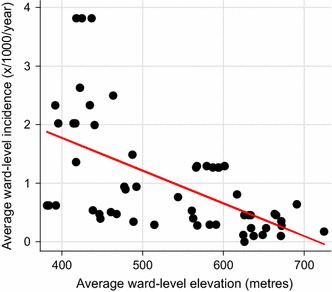
Scatter plot of average reported malaria incidence per study ward between 2010 and 2015 and average study ward elevation. Elevation in metres from sea level. Slope: −0.006, r: −0.58

**Fig. 6:**
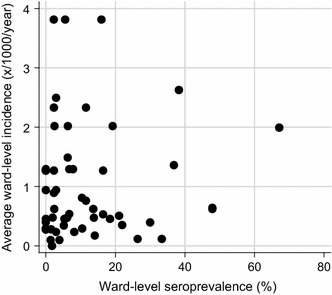
Scatter plot of average reported malaria incidence per study ward between 2010 and 2015 and study ward seroprevalence. Ward-level seroprevalence corresponds to the percentage of sampled seropositive households in each ward

Very low malaria incidence between 2010 and 2015, averaging 0.95 cases per 1000 population, was reported across the whole study region, with values ranging between 0.1 and 3.8 per 1000 population among individual study site wards. Moreover, historical malaria incidence was heterogenous across the study region as wards, that reported <one case per 1000 population, were often situated adjacent to each other (Fig. 1). Variation in elevation was also observed across the study site, with household elevation ranging from 363 to 815 m. Average study ward historical incidence and elevation was inversely associated (slope: −0.006, r: −0.58), highlighting lower incidence at higher ground elevation (Fig. 5). No statistically significant linear relationship was observed between historical malaria incidence and ward-level *P. falciparum* seroprevalence (Fig. 6).

## Discussion

This cross-sectional, baseline survey, nested within an ongoing targeted IRS trial [26], aimed to investigate the transmission dynamics, spatial distribution and risk factors associated with *P. falciparum* exposure within the study area using serological markers, the results of which are to be used to both inform elimination efforts in South Africa and assess whether sero-epidemiological methods have the potential to be routinely incorporated into elimination programmes.

The overall low malaria risk in the northeast border regions of South Africa has been well documented [30], however until now no detailed population-based assessment of transmission intensity had been conducted. This first sero-epidemiological, cross-sectional study investigating exposure to malaria, estimated a *P. falciparum* SCR of 0.012 per year, which translates to only 1.2/100 sampled participants becoming seropositive to malaria every year. This result suggests a low force of malaria infection in this region of South Africa, which is expected given the very low reported incidence in the area of approximately one case per 1000 per year and is similar to serological findings in nearby Swaziland [31]. Although a SCR revealed overall MTI to be low across the entire study area, variations in malaria incidence among study site wards and contrasting SCRs between geographically distinct geographical regions highlight prominent heterogeneity of MTI in this area of South Africa. Similar spatial patterns in malaria transmission have been observed in other low transmission settings [5, 18, 21, 24, 32], although few have been conducted in very low endemic settings. It is therefore important to note that while measures of MTI, including SCRs, EIRs and incidence data, recorded over vast geographical areas provide a useful overall picture of malaria transmission, these measures do not necessarily reflect transmission intensity at the micro-epidemiological level. In the Ba-Phalaborwa study site for instance, an area that spans approximately 10 × 20 km, most serological evidence of elevated transmission intensity is focused in the northern half of this region. This suggests a MTI value across this area would not be representative of true transmission at ground level.

Spatial clusters of *P*. *falciparum* seropositive households and elevated age-adjusted *P. falciparum* antibody responses suggest malaria transmission is not only heterogeneous across this study region, but concentrated in malaria hotspots. Hotspots are defined as areas which experience a significantly higher burden of malaria compared to the surrounding vicinity and may act as geographical reservoirs of the disease [6, 15, 29, 33]. To further characterize identified hotspots in the study region, malaria seroprevalence in study participants aged 5 years and under was used as a proxy for detecting recent malaria exposure. Households with seropositive individuals aged ≤5 years were predominately located within hotspots of seropositive households containing all ages, implying these malaria hotspots are likely persistent contributors to malaria transmission and not representative of historical exposure. It should be noted however that many OD values among seropositive children are only just above the seropositive cut-off value. Moreover, elevated antibody responses in children could be attributed to hypergammaglobulinaemia [34], a process in which antibody production is accelerated due to immunological exposure to other antigens.

Previous studies have identified numerous favourable abiotic and biotic conditions that are believed to facilitate the existence of malaria hotspots, ranging from mosquito breeding site proximity to poor health care practices [5, 35–37]. In this study, poor formal education, a likely proxy for low socio-economic status (SES), was associated with increased malaria exposure. This association is consistent with the rationale that poor quality housing better enables mosquitoes to infect inhabitants [36, 38]. Across the study region, increasing elevation was inversely associated with increasing historical malaria incidence, which is expected given lower temperatures at higher attitudes are believed to impede parasite sporogony in the mosquito gut leading to reduced transmission [39]. The association between elevation and malaria seroprevalence in this study however was more ambiguous, as no declining trend in malaria seroprevalence with increasing elevation was observed. This observation may suggest other factors, aside from elevation, including malaria importation, and successful control efforts have greater impacts on transmission dynamics in lower transmission settings, although this requires further validation. Despite the ambiguity, it should be noted that both historical malaria incidence and malaria seroprevalence was reduced, although not significantly, above altitudes of 650 m.

In this survey, historical malaria incidence represents symptomatic cases that reported to health facilities between 2010 and 2015 while seroprevalence reflects those who have been exposed to the *P. falciparum* parasite, potentially decades prior. Thus, the observed non-linear relationship between increasing average ward-level incidence and increasing ward-level percentage seroprevalence could be explained by this study detecting previously exposed and/or asymptomatic individuals who may not have reported to health facilities between 2010 and 2015. If this discordance between exposure and incidence is confirmed in more extensive surveys, it raises a question for targeted vector control strategies: should these be directed at foci of higher case incidence or should they be directed at foci of higher exposure to malaria?

Following antigen stratification, seroprevalence and overall seroconversion was higher for PfAMA-1 compared to PfMSP-1_19_, a result shown elsewhere and thought to be the result of contrasting immunogenicities between the two antigens [7, 32]. This disparity may account for the observed spatial discordance in age-adjusted antibody responses towards each antigen in certain study site areas. In the Ba-Phalaborwa study site for instance, a cluster of elevated PfAMA-1, but not PfMSP-1_19_, antibody responses was detected. This discordance would explain why seroprevalence was lower in the Ba-Phalaborwa study site and highlights the importance of using both these serological markers to more effectively characterize malaria exposure, particularly in low endemic settings where antibody responses are low. Nevertheless, given antigenic discordance has been observed elsewhere [40], this puts into question whether these two serological markers, alone, truly characterize malaria exposure correctly. Fortunately, identifying novel serological markers that accurately represent recent exposure to malaria is an area of continued investigation [41].

### Limitations

Oversampling of unemployed adult females in this study may have resulted in results that are not fully representative of the study population. Most notable is recent cross-border travel not being significantly associated with malaria exposure, despite a higher proportion of reported travellers being seropositive to malaria. This is attributed to few reports of travel, likely due to the lack of adult males surveyed who are thought to undertake more cross-border travel than adult females [42]. The low sampling rate of males may also have led to the finding of weak evidence that seroprevalence is higher in females than in males. Oversampling is likely a result of survey timing, as most surveys were conducted during weekdays when children and employed adult males were often absent.

## Conclusions

This study verifies that serology is effective in characterizing malaria transmission dynamics, even in a very hypo-endemic setting where antibody responses to malaria are low. Therefore, by routinely incorporating sero-epidemiological practices into elimination programmes, this offers countries embarking on malaria elimination the opportunity to both characterize and monitor malaria transmission. This survey, conducted in the northeastern region of South Africa, revealed MTI is both low and spatially heterogenous. This reinforces the notion that malaria elimination in South Africa is achievable, provided amendments to existing methods of control are adopted. Such prominent transmission heterogeneity suggests widespread control interventions are likely to have varying degrees of success while targeting interventions has the potential to be a more appropriate, cost-effective and sustainable strategy for eliminating malaria. However, whether this strategy can cope with malaria epidemics remains unknown, and should be taken into consideration when South Africa pilots a new enhanced surveillance and response strategy in selected malaria hotspots.

## Additional files

**Additional file 1.** Box plots of antibody responses, in the form of optical density (OD) values, for both PfAMA-1 and PfMSP-1_19_ among different ages/age bands across the entire study site. Hollow black circles represent outlier OD responses. Red lines correspond to seropositive cut-offs, which equate to 0.067 for PfAMA-1 and 0.103 for PfMSP-1_19_.

**Additional file 2.** Age-seroprevalence to either PfAMA-1 or PfMSP-1_19_ among sampled participants of both Ba-Phalaborwa and Bushbuckridge. Red triangles: observed age-seroprevalence; solid lines: predicted seroprevalence; dotted lines: predicted seroprevalence upper and lower 95% confidence intervals.

**Additional file 3.** Demographic and risk factors associated with seroprevalence to either PfAMA-1 and PfMSP-1_19_.

## Abbreviations

AOR: adjusted odds ratio
CI: confidence interval
EIR: entomological inoculation rate
DDT: dichlorodiphenyltrichloroethane
ELISA: enzyme-linked immunosorbent assay
IRS: indoor residual spraying
KAP: knowledge, attitudes and practice
MTI: malaria transmission intensity
OD: optical density
OR: odds ratio
PfAMA-1: *Plasmodium falciparum* apical membrane antigen-1
PfMSP-1_19_: *Plasmodium falciparum* merozoite surface protein-1_19_
SCR: seroconversion rate
SES: socio-economic status
WHO: World Health Organization
