# Supplementary figures and images for: Serology reveals heterogeneity of Plasmodium falciparum transmission in northeastern South Africa: implications for malaria elimination
Source: Malar J. 2017 Jan 26;16:48. doi: 10.1186/s12936-017-1701-7 (PMC5270351; doi:10.1186/s12936-017-1701-7)

**
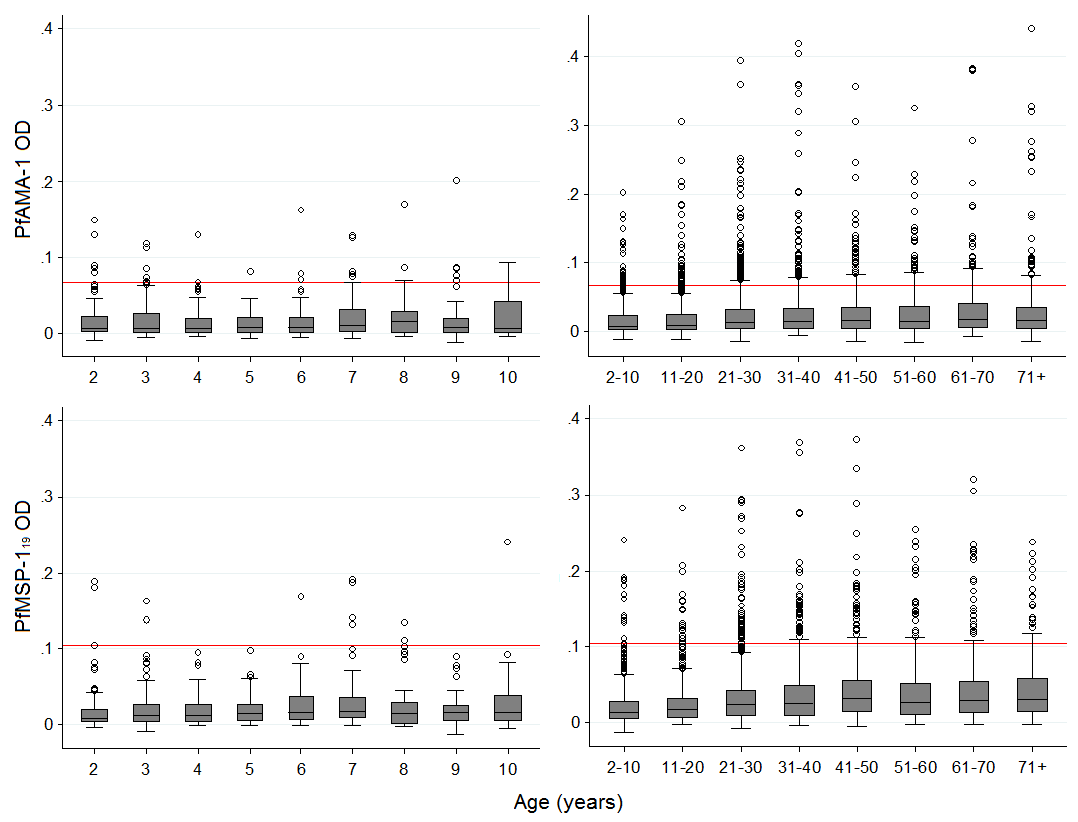
**

Supplement: Supplementary file 1 — Additional file 1. Box plots of antibody responses, in the form of optical density (OD) values, for both PfAMA-1 and PfMSP-119 among different ages/age bands across the entire study site. Hollow black circles represent outlier OD responses. Red lines correspond to seropositive cut-offs, which equate to 0.067 for PfAMA-1 and 0.103 for PfMSP-119. [file 12936_2017_1701_MOESM1_ESM.docx]

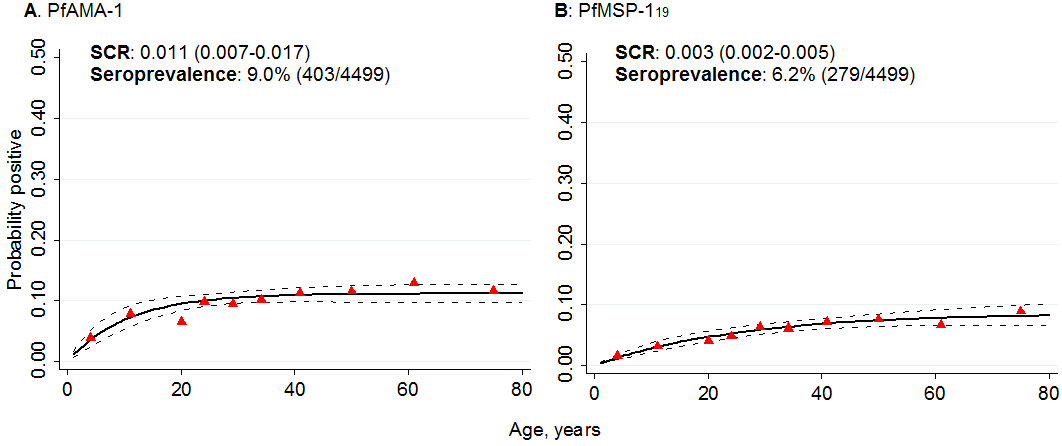

Supplement: Supplementary file 2 — Additional file 2. Age-seroprevalence to either PfAMA-1 or PfMSP-119 among sampled participants of both Ba-Phalaborwa and Bushbuckridge. Red triangles: observed age-seroprevalence; solid lines: predicted seroprevalence; dotted lines: predicted seroprevalence upper and lower 95% confidence intervals. [file 12936_2017_1701_MOESM2_ESM.docx]
